# Analysis of factors influencing prevalence and malignancy of thyroid nodules in various iodine uptake areas

**DOI:** 10.3389/fendo.2024.1451911

**Published:** 2024-11-07

**Authors:** HongLei Xie, HaoWen Pan, TingTing Qian, Xin Hou, Meng Zhao, WenJing Che, WeiDong Li, Xian Xu, Yue Su, Jia Li, ZongYu Yue, ZeXu Zhang, Peng Liu

**Affiliations:** ^1^ Center for Endemic Disease Control, Chinese Center for Disease Control and Prevention, Harbin Medical University, Harbin, China; ^2^ Institute for Endemic Disease Prevention and Control, Yun nan Institute of Endemic Diseases Control and Prevention, Dali, China; ^3^ Institute for Endemic Disease Prevention and Control, Anhui Provincial Center for Disease Control and Prevention, Hefei, China; ^4^ Key Lab of Etiology and Epidemiology, Education Bureau of Heilongjiang Province (23618504) & Ministry of Health, Heilongjiang Provincial Key Laboratory of Trace Elements and Human Health, Harbin, China

**Keywords:** thyroid nodules, prevalence, nodule grading, risk factor, iodine

## Abstract

**Background:**

The prevalence of thyroid nodules (TNs) has been increasing rapidly. However, little is known about the drivers of its high prevalence and tendency of malignancy. This study aimed to analyze the factors influencing the prevalence and malignancy of TNs in the adult population.

**Methods:**

A multi-stage stratified cluster random sampling was used to conduct a cross-sectional survey of the population in different iodine uptake areas in Anhui Province. The areas with deficient, adequate, and excess iodine intake were grouped according to population's urine iodine. A questionnaire, laboratory examination and ultrasound diagnosis were conducted on the participants. Nodules were diagnosed and distinguished using ultrasonography. Spearman rank correlation, random forest importance ranking, ROC curve, and unconditional binary logistic regression analyses were used to screen for risk factors.

**Results:**

A total of 1,697 participants (539 males and 1,158 females) aged 18–60 years were included, 355 of whom were diagnosed with TN. The prevalence of TNs was 20.9% and varied in different areas, with 21.9%, 25.8%, and 18.0% in the iodine deficient, adequate, and iodine excess areas, respectively. The prevalence of TNs in females was significantly higher than that in males (24.5% vs. 13.2%) and it increased with age. Female sex (OR, 1.67 [1.21–2.30]), old age (>41 years, OR, 2.00 [1.14, 3.50]) and smoking were risk factors for the development and deterioration of TNs.

**Conclusions:**

Patients with TNs should exercise caution when consuming goitrogens and adhere to a scientifically balanced diet. Given the high incidence of TNs in field setting, it is necessary to raise public health awareness among residents and perform regular thyroid ultrasound screening to facilitate early detection and treatment.

## 1 Introduction

Thyroid disease is a common endocrine condition. Approximately one in five individuals experience a thyroid functional or structural aberration during their lifetime (1). Among the thyroid diseases, those related to thyroid nodules (TNs) are the most prevalent. Imaging technique-related advancements contributed to a recent TN prevalence, leading to an increase in the detection rate (2, 3). Detection rates reportedly ranged between 19% and 67% in randomly sampled populations (4), with thyroid cancer accounting for 5–15% of the cases (5). Moreover, thyroid disease prevalence increases with age, with higher rates in women and older adults.

Research among adults in iodine-adequate areas revealed a strong association between metabolic syndrome and TNs prevalence risk (6). However, no study in China has yet described how iodine intake level (adequate intake vs. excessive iodine) influences TNs prevalence, the risk factors for their development, or their progression to malignancy after grading. The high prevalence of TNs cannot be attributed solely to increased iodine intake; other factors such as diet, obesity, and alcohol consumption might also affect TNs prevalence (7–9). Therefore, investigating factors other than iodine that could contribute to the high goiter and TNs incidence would be necessary (10, 11).

Substances that affect thyroid hormone synthesis and secretion, which cause goiter in addition to iodine deficiency (12), are known as goitrogenic substances (**Supplementary Figure 1**). Goitrogenic substances in food have two or more synergistic effects on human thyroid function and can be divided into two categories (13). First, vegetables such as cabbage, turnip, corn, and cassava produce large amounts of thiocyanate or thiocyanate compounds in the body, which primarily inhibit the iodine concentration mechanism of the thyroid (14). In addition, a class of disulfide compounds found in garlic and onion results in thiourea-like effects, inhibiting the absorption of iodine by the thyroid during periods of low iodine consumption (15). Flavonoids, such as those in millet, sorghum, and walnut, are polyhydroxy phenolic compounds with C6-C3-C6 structures, which alter the synthesis pattern of thyroid hormones (16, 17).

Numerous factors affecting the pathogenesis of TNs have not yet been fully identified. Environmental factors, diet, smoking, stress, radiation, genetic predispositions, and other factors may trigger the onset of TNs. Therefore, this study focuses on the influence of various factors on the incidence and malignant transformation of TNs in the population. Specifically, this study aimed to investigate the factors influencing the high incidence of TNs, and to predict the risk of benign and malignant TNs based on ultrasound characteristics.

## 2 Materials and methods

### 2.1 Ethics statement

The collection, preservation, utilization, and external provision of human genetic resources involved in this study were carried out in accordance with the relevant provisions of the Regulations of the People's Republic of China on the Management of Human Genetic Resources. This study was conducted in accordance with the Declaration of Helsinki and ethical approval was granted by the Ethics Committee of China Medical University (approval number: YS2023YFC2500017). Verbal informed consent was obtained from all participants prior to inclusion.

### 2.2 Study participants

A cross-sectional epidemiological survey was conducted in five areas of Anhui Province from January 2022 to May 2023. The areas were Shaozhuang Village, Huangkou Town, Xiaoxian County, Suzhou; Liwa Village, Gaoyue Town, Duji, Huaibei, Yuqiao Village, Zhongxing Town, Guzhen Town, Bengbu, Qinglong Village, Qinglong Town, Xiaoxian County, Suzhou; and Tongcheng, Anqing. A total of 2,190 adults from five regions were recruited in this study. Women who were pregnant or lactating (n=114), participants with incomplete data (n=246), and those taking iodine-containing drugs, such as Cydiodine buccal tablets and Amiodarone hydrochloride in the past week (n=50), were excluded from the study. Additionally, 65 individuals who had recently consumed food containing high iodine levels and 18 patients with a history of thyroid disease were excluded. A total of 1,697 participants aged 18–60 years (comprising 539 males and 1,158 females), were included in the final analysis. The patient selection flowchart is shown in **Supplementary Figure 2**.

### 2.3 Measurements

A team of staff trained by the Institute of Iodine Deficiency Disorders Control, Endemic Disease Control Center of China CDC conducted face-to-face interviews at community health service centers or township health centers at each survey site. Personal information of each participant, including general information (name, sex, age, height, weight, and fertility history), disease and medication history, lifestyle habits (smoking, alcohol consumption, types of salt consumed, and frequency of consumption of goitrogenic substances), and family history of thyroid diseases, were collected through questionnaires. Subsequently, thyroid ultrasound was performed to measure and obtain the transverse and anteroposterior diameters of each thyroid lobe, as well as the anteroposterior isthmus thickness. If TNs were found, their location, number, size, nature, echo, boundary, and vascularity were recorded. Multiple nodules were measured to determine their maximum diameter. A random urine sample was collected to determine iodine concentration in the urine.

### 2.4 Evaluation criteria

TNs were diagnosed using B-ultrasound. The same high-resolution portable (Mindray 8) thyroid ultrasound instrument (ESAOTE MyLab30 model, 7.5 MHz linear transducer; Shenzhen Mindray Biomedical Electronics Co., Ltd., Shenzhen, China) with intensive training by professional sonographers at the Fourth Affiliated Hospital of Harbin Medical University was used for all participants. Based on these thyroid B-ultrasound results, a doctor graded the TNs using thyroid imaging reporting and data system (TI-RADS) classification. According to the 2017 American College of Radiology TI-RADS (18) and the 2020 Thyroid Chinese Guidelines for Malignant Risk Stratification of Nodules by Ultrasound (C-TIRADS) (19), TNs were classified into benign nodules (TI-RADS 3 and below) and suspected malignant nodules (TI-RADS 4a, 4b, and 4c). All TNs were graded according to the location, orientation, edge, sound halo, structure, echo texture, and focal strong echo. Grade 2 benign nodules were used as controls to explore the factors influencing TN grade in different iodine intake regions. The cutoffs for nutritional iodine levels were derived using the median urinary iodine level of the general adult population. Iodine “deficiency” was described as urinary iodine <100 µg/L, “adequate” as 100–300 µg/L, and “excess” as ≥300 µg/L.

### 2.5 Statistical analysis

All the collected data were entered and sorted in Excel 2019. R software (version 4.3.2. Microsoft Corporation, United States) was used for statistical analyses of data. The Chi-square test was used to compare rates. Kruskal–Wallis H or Mann–Whitney U rank sum tests were used to compare two independent samples of non-normally distributed data. Logistic regression analysis was used to analyze risk factors. Related risk factors were screened by multivariate analysis using non-conditional binary logistic regression analysis. Statistical significance was set at P < 0.05.

## 3 Results

### 3.1 Analysis of related factors of thyroid volume size

#### 3.1.1 A linear regression model of thyroid volume was established

A total of 1,697 participants were included. A linear regression model on thyroid volume was established, and independent variables that had impact on thyroid volume were subsequently screened by step-based regression. The step-based regression results were shown in **Table 1**. Urinary iodine grouping, sex, body mass index (BMI) classification, and eating times of cabbage have significant effects on thyroid volume (**Table 1**). When other independent variables remained constant, the urinary iodine group changed from adequate to above adequate iodine, and the total thyroid volume increased by 0.086202 units on average. When other independent variables were held constant, women's total thyroid volume decreased by 0.16 units compared to that of men. Overweight increased the total volume of the thyroid by 0.09 units compared to underweight. Obesity increased the total thyroid volume by 0.15 units compared with underweight. When other independent variables were held constant, eating cabbage 1–6 times per week increased the total thyroid volume by 0.06 units compared with not eating cabbage at all.

**Table 1:** Thyroid volume stepwise regression results.

Index	Level	*β*	SE	OR (95%CI)	*p*
Urinary iodine	Iodine deficiency				
	Adequate	0.09	0.03	1.09 (1.03, 1.15)	0.002
	Iodine excess	0.01	0.02	1.01 (0.96, 1.05)	0.764
Sex	Male				
	Female	-0.16	0.02	0.86 (0.82, 0.89)	<0.001
Age (years)	21–30				
	31–40	0.05	0.04	1.05 (0.96, 1.14)	0.266
	41–50	0.01	0.04	1.01 (0.93, 1.09)	0.835
	51–60	-0.03	0.04	0.97 (0.90, 1.05)	0.440
BMI	Underweight				
	Normal weight	-0.03	0.11	0.97 (0.78, 1.20)	0.763
	Overweight	0.09	0.02	1.09 (1.04, 1.15)	<0.001
	Obesity	0.15	0.03	1.16 (1.10, 1.22)	<0.001
Family history of thyroid diseases	No				
	Yes	0.10	0.05	1.11 (1.00, 1.24)	0.057
Frequency of	Infrequent				
cabbage consumption	1–6 times a week	0.06	0.02	1.06 (1.01, 1.11)	0.026
	1–2 times a day	0.05	0.05	1.05 (0.96, 1.15)	0.297
Eating disulfide-containing foods	Infrequent				
	1–6 times a week	0.01	0.04	1.01 (0.94, 1.08)	0.874
	1–2 times a day	-0.04	0.04	0.96 (0.89, 1.04)	0.319

### 3.2 Analysis of factors related to TNs

#### 3.2.1 General characteristics of participants according to the presence of TNs

The prevalence of TNs among the 1,697 participants was 20.9% (24.5% in females and 13.2% in males). A statistically significant differences were observed in urinary iodine levels, age, sex, smoking status, and history of alcohol consumption in the past year between patients with and without TNs (**Table 2**). After further group comparisons, a statistically significant difference was found between the areas with excess and adequate levels of urine iodine.

**Table 2:** Comparative analysis of clinical and demographic factors according to the presence of thyroid nodules.

Index		Total	Thyroid nodule	No thyroid nodule	χ^2^	P
n		1697	355	1342		
Urinary iodine					8.605	<0.05
	Iodine deficiency	645	141 (21.9%)	504 (78.1%)		
	Adequate	314	81 (25.8%)	233 (74.2%)		
	Iodine excess	738	133 (18.0%)	605 (82.0%)		
Sex					28.654	<0.001
	Male	539	71 (13.2%)	468 (86.8%)		
	Female	1158	284 (24.5%)	874 (75.5%)		
Age (years)					21.155	<0.001
	21–30	131	16 (12.2%)	115 (87.8%)			
	31–40	288	42 (14.6%)	246 (85.4%)			
	41–50	447	90 (20.1%)	357 (79.9%)			
	51–60	831	207 (24.9%)	624 (75.1%)	
Occupation					0.453	0.797	
	Light labor	179	34 (19.0%)	145 (81.0%)		
	Heavy labor	1274	269 (21.1%)	1005 (78.9%)	
	Other	244	52 (21.3%)	192 (79.7%)	
BMI					0.907	0.824
	Underweight	14	2 (14.3%)	12 (85.7%)			
	Normal weight	541	109 (20.1%)	432 (79.9%)	
	Overweight	702	153 (21.8%)	549 (78.2%)	
	Obesity	440	91 (20.7%)	349 (79.3%)	
Alcohol consumption					31.606	<0.001	
	Nondrinker	1206	295 (24.5%)	911 (75.5%)			
	Drinker	491	60 (12.2%)	431 (87.8%)			
Smoking status					11.395	<0.01	
	Nonsmoker	1376	310 (22.5%)	1066 (77.5%)			
	Smoker	321	45 (14.0%)	276 (86.0%)			
Family history of thyroid diseases					0.127	0.722	
	No	1640	342 (20.6%)	1298 (79.1%)			
	Yes	57	13 (22.8%)	44 (77.2%)			
Family genetic disease history					2.266	0.132	
	No	1545	133 (8.6%)	1229 (91.4%)			
	Yes	152	39 (25.7%)	133 (74.3%)			
Radiation history					0.461	0.497	
	No	1568	325 (20.7%)	1243 (79.3%)			
	Yes	129	30 (23.3%)	99 (76.7%)			
Salt intake					5.298	0.071	
	Moderate	709	133 (18.8%)	576 (81.2%)			
	Light	603	144 (23.9%)	459 (76.1%)			
	Heavy	385	78 (20.3%)	307 (79.7%)			

BMI, body mass index.

#### 3.2.2 Multivariate regression model analysis of factors associated with the presence of TNs

Logistic regression results showed that sex, age, alcohol consumption in the past year, amount of radishes consumed, and amount of turnips consumed were statistically correlated with the odds of developing TNs when adjusted for urinary iodine. Female sex was a risk factor for developing TNs, and women were 1.67 times more likely to have nodules than men. Participants who drank alcohol were 0.59 times less likely to develop TNs than those who did not drink alcohol. Eating radishes and turnips have been identified as protective factors against TNs. Those who ate radishes 1–2 times a day were 0.47 times less likely to develop TNs than those who did not. Those who ate turnips 1–6 times per week were 0.58 times less likely to develop TN than those who did not eat turnips at all (**Supplementary Figure 3**).

A receiver operating characteristic (ROC) curve was constructed to represent the results of the stepwise regression model. As shown in **Supplementary Figure 4**, the area under the curve (AUC) was 0.649, suggesting that the predictive ability of the model was slightly better than that of a random guess (AUC=0.5). The stepwise regression model identified urinary iodine, sex, age, alcohol consumption in the past year, consumption of radish and turnip, and TNs as significant predictors of thyroid volume. These findings highlight the important role of these factors in predicting the presence or absence of nodules (**Supplementary Figure 4**).

### 3.3 Analysis of factors related to single and multiple TNs

#### 3.3.1 Comparative analysis of participant characteristics stratified by number of TNs

A total of 355 participants with nodules were identified, of whom 245 (69%) had single nodules and 110 (31%) had multiple nodules. Bivariate analysis of the general characteristics showed no statistical differences between the two groups (**Table 3**).

**Table 3:** Demographic characteristics of participants according to the number of thyroid nodules.

Index		Total	Single nodule	Multiple nodules	P
n		355	245 (69.0%)	110 (31.0%)	
Urinary iodine					0.546
	Iodine deficiency	141	102 (73.8%)	39 (26.2%)	
	Adequate	81	54 (66.7%)	27 (33.3%)	
	Iodine excess	133	89 (67.0%)	44 (33.0%)	
Sex					0.115
	Male	71	55 (77.5%)	16 (22.5%)	
	Female	284	190 (66.9%)	94 (33.1%)	
Age (years)					0.266
	21–30	16	10 (62.5%)	6 (37.5%)	
	31–40	42	26 (61.9%)	16 (38.1%)	
	41–50	90	69 (76.7%)	21 (23.3%)	
	51–60	207	140 (67.6%)	67 (32.4%)	
Occupation					0.984
	Light labor	34	23 (67.6%)	11 (32.4%)	
	Heavy labor	269	186 (69.1%)	83 (30.9%)	
	Other	52	36 (69.2%)	16 (30.8%)	
BMI					0.800
	Underweight	2	1 (50.0%)	1 (50.0%)	
	Normal weight	109	74 (67.9%)	35 (32.1%)	
	Overweight	153	104 (68.0%)	49 (32.0%)	
	Obesity	91	66 (72.5%)	25 (27.5%)	
Alcohol consumption					0.344
	Non-drinker	295	200 (67.8%)	95 (32.2%)	
	Drinker	60	45 (75.0%)	15 (25.0%)	
Smoking status					0.125
	Nonsmoker	310	209 (67.4%)	101 (32.6%)	
	Smoker	45	36 (80.0%)	9 (20.0%)	
Family history of thyroid diseases					0.773
	No	342	237 (69.3%)	105 (30.1%)	
	Yes	13	8 (61.5%)	5 (38.5%)	
Radiation history					0.903
	No	325	224 (69.0%)	101 (31.0%)	
	Yes	30	21 (70.0%)	9 (30.0%)	
Salt intake					0.558
	Moderate	133	96 (72.2%)	37 (27.8%)	
	Light	144	98 (68.1%)	46 (31.9%)	
	Heavy	78	51 (65.4%)	27 (34.6%)	

#### 3.3.2 Multivariate regression model analysis of factors associated with the number of TNs

**Figure 1:**
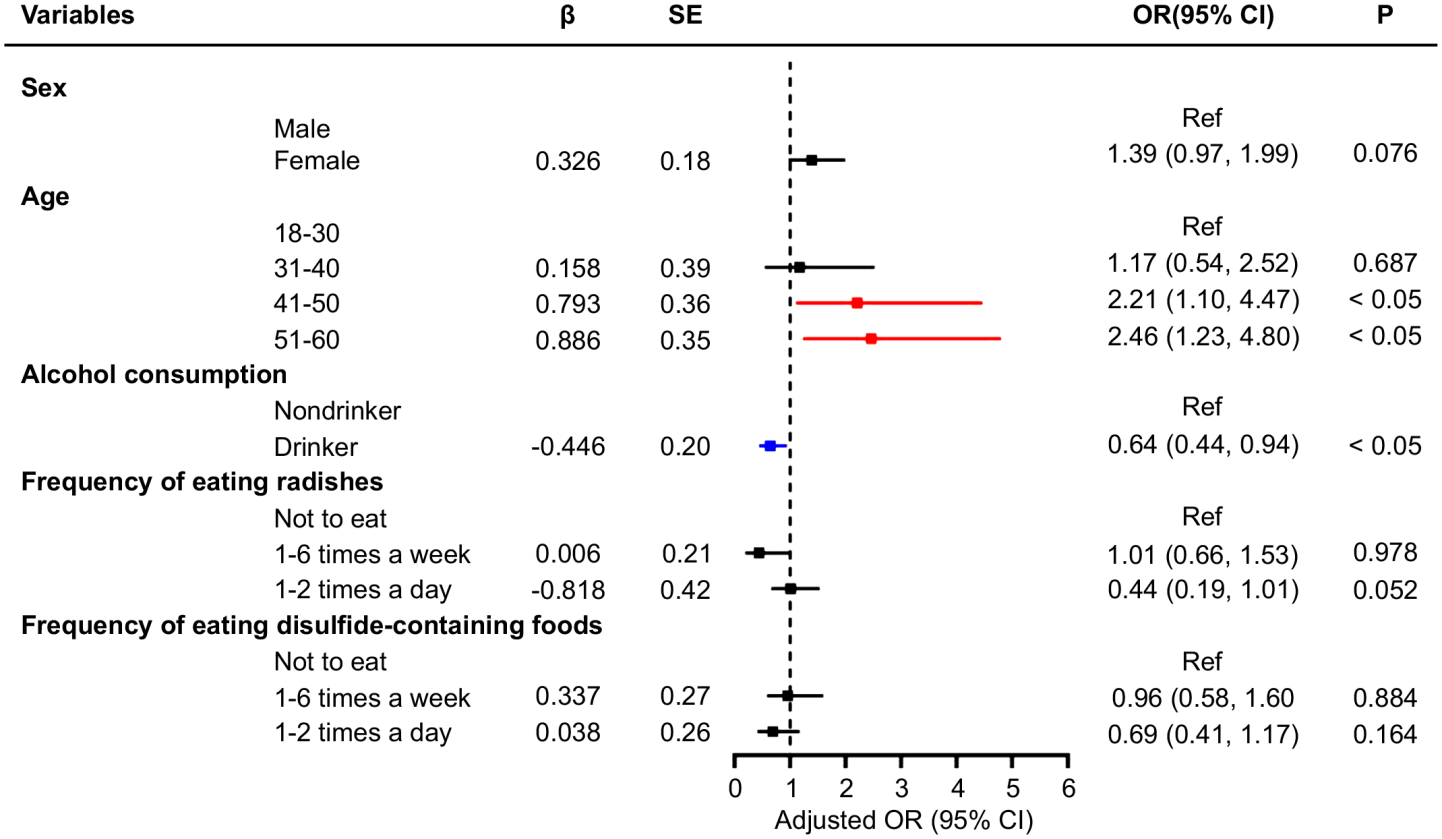
Multivariate logistic regression analysis of factors associated with single thyroid nodules.

**Figure 2:**
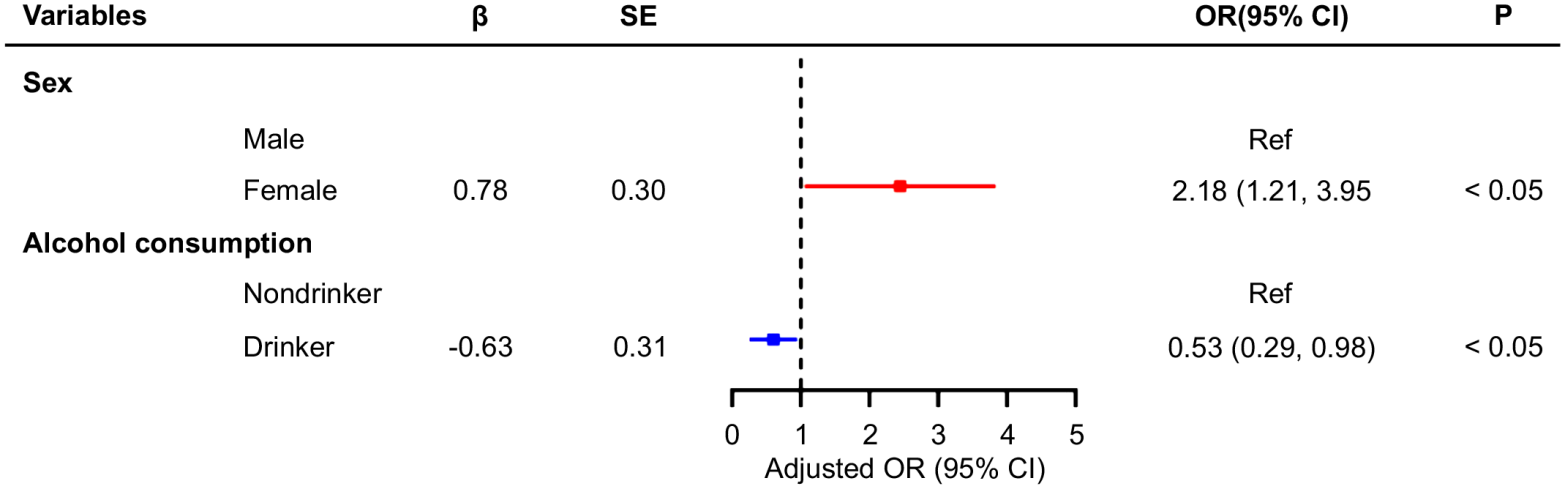
Multivariate logistic regression analysis of factors associated with multiple thyroid nodules.

The number of TNs differed between those who consumed food containing disulfide and those who did not. Participants who ate disulfide-containing foods 1–2 times a day were 0.38 times less likely to develop multiple nodules than those who did not (**Table 4**). In addition, logistic regression analysis was performed for participants with single and multiple nodules in the general population (**Figures 1**, **2**), and showed that age and alcohol consumption in the past year were significantly associated with the risk of developing TNs when controlling for urinary iodine.

**Table 4:** Logistic regression analysis of factors influencing the number of thyroid nodules.

Variable	Level	β	SE	OR (95% CI)	P
Sex	Male			Ref	
	Female	0.530	0.31	1.70 (0.92, 3.14)	0.090
Eating disulfide-containing food	Infrequent			Ref	
	1–6 times a week	-0.361	0.25	0.70 (0.43, 1.13)	0.140
	1–2 times a day	-0.971	0.49	0.38 (0.14, 0.99)	<0.05

**Figure 3:**
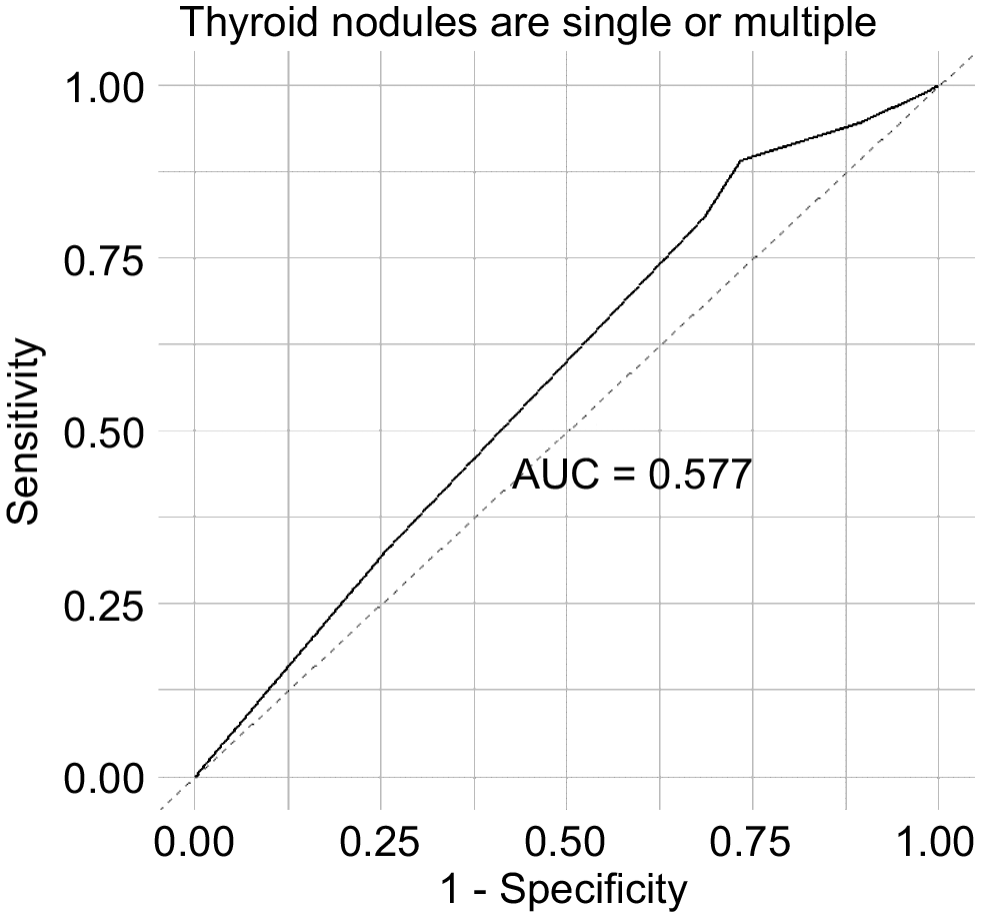
ROC curves of variables affecting the number of thyroid nodules.

Participants who drank alcohol in the last year were 0.64 times more likely to develop a single nodule than those who had not. Compared with individuals aged 18–30 years, those aged 41–50 and 51–60 years were 2.21 and 2.46 times more likely to develop a single TNs, respectively. When controlling for urinary iodine levels, the factors influencing the development of multiple TNs were sex and alcohol consumption in the past year. Women were 2.18 times more likely to have multiple nodules than men, whereas participants who drank alcohol were 0.53 times less likely to develop multiple nodules than those who did not (**Figure 2**). An ROC curve was plotted to represent the results of the stepwise regression model. As shown in **Figure 3**, the AUC was 0.577, indicating that the model had a poor ability to predict the occurrence of nodules and performed similarly to random guessing. According to the stepwise regression results, the overall prediction of the model was inadequate, and the consumption of disulfide did not have a significant effect on TNs (**Figure 3**).

### 3.4 Analysis of factors influencing TNs grading

#### 3.4.1 Distribution of demographic characteristics based on TNs grade

Among 355 participants with TNs, 116 were examined using ultrasound and classified using the TI-RADS classification. The remaining 239 cases were not analyzed because of small nodules and insufficient B-ultrasonography findings for accurate classification. The distribution of the ultrasonographic TI-RADS classifications were as follows: 20 cases of grade 2, 52 cases of grade 3, 28 cases of grade 4a, 12 cases of grade 4b, and 4 cases of grade 4c. No statistical difference was observed in each index (**Table 5**).

**Table 5:** Distribution of demographic characteristics according to thyroid nodule grading.

Variable	Level	Grade 2 (n=20)	Grade 3 (n=52)	Grade 4a (n=28)	Grade 4b (n=12)	Grade 4c (n=4)	P
Urinary iodine (%)	Iodine deficiency	4 (20.0)	23 (44.2)	12 (42.9)	5 (41.7)	3 (75.0)	0.102
	Adequate	5 (25.0)	7 (13.5)	10 (35.7)	2 (16.7)	1 (25.0)	
	Iodine excess	11 (55.0)	22 (42.3)	6 (21.4)	5 (41.7)	0 (0.0)	
Sex (%)	Male	7 (35.0)	7 (13.5)	6 (21.4)	0 (0.0)	0 (0.0)	0.071
	Female	13 (65.0)	45 (86.5)	22 (78.6)	12 (100.0)	4 (100.0)	
Age (%)	18–30	4 (20.0)	4 (7.7)	4 (14.3)	0 (0.0)	0 (0.0)	0.426
	31–40	4 (20.0)	6 (11.5)	3 (10.7)	1 (8.3)	2 (50.0)	
	41–50	3 (15.0)	12 (23.1)	8 (28.6)	3 (25.0)	0 (0.0)	
	51–60	9 (45.0)	30 (57.7)	13 (46.4)	8 (66.7)	2 (50.0)	
BMI (%)	Underweight	7 (35.0)	15 (28.8)	5 (17.9)	2 (16.7)	1 (25.0)	0.725
	Normal weight	1 (5.0)	1 (1.9)	0 (0.0)	0 (0.0)	0 (0.0)	
	Overweight	7 (35.0)	26 (50.0)	16 (57.1)	5 (41.7)	1 (25.0)	
	Obesity	5 (25.0)	10 (19.2)	7 (25.0)	5 (41.7)	2 (50.0)	
Occupation (%)	Light labor	1 (5.0)	4 (7.7)	2 (7.1)	1 (8.3)	0 (0.0)	0.996
	Heavy labor	15 (75.0)	40 (76.9)	21 (75.0)	8 (66.7)	3 (75.0)	
	Other	4 (20.0)	8 (15.4)	5 (17.9)	3 (25.0)	1 (25.0)	
Family history of thyroid diseases (%)	No	19 (95.0)	50 (96.2)	28 (100.0)	12 (100.0)	4 (100.0)	0.743
	Yes	1 (5.0)	2 (3.8)	0 (0.0)	0 (0.0)	0 (0.0)	
Radiation history (%)	No	19 (95.0)	50 (96.2)	27 (96.4)	12 (100.0)	4 (100.0)	0.946
	Yes	1 (5.0)	2 (3.8)	1 (3.6)	0 (0.0)	0 (0.0)	
Alcohol consumption (%)	Non-drinker	15 (75.0)	43 (82.7)	22 (78.6)	12 (100.0)	4 (100.0)	0.34
	Drinker	5 (25.0)	9 (17.3)	6 (21.4)	0 (0.0)	0 (0.0)	
Smoking status (%)	Nonsmoker	18 (90.0)	49 (94.2)	23 (82.1)	12 (100.0)	4 (100.0)	0.275
	Smoker	2 (10.0)	3 (5.8)	5 (17.9)	0 (0.0)	0 (0.0)	
Salt intake (%)	Moderate	4 (20.0)	21 (40.4)	12 (42.9)	6 (50.0)	1 (25.0)	0.11
	Light	8 (40.0)	18 (34.6)	14 (50.0)	5 (41.7)	3 (75.0)	
	Heavy	8 (40.0)	13 (25.0)	2 (7.1)	1 (8.3)	0 (0.0)	

#### 3.4.2 Factors influencing TNs grading

The stepwise regression analysis revealed a significant differences in the degree of nodule grading between females and males (**Table 6**), with females having a 13.19-fold higher probability of higher nodule grading compared to males. Furthermore, the probability of obtaining a higher nodule grading in overweight and obese individuals was 2.48 and 3.88 times that of underweight individuals, respectively. Smoking was 4.84 times more likely to result in a higher grade of nodules than not smoking. Heavy salt intake was 0.13 times more likely to result in higher-grade nodules than moderate salt intake. Compared with participants who did not consume cabbage in their diet, eating cabbage 1–6 times weekly and 1–2 times daily were 4.64 and 7.57 times more likely to result in higher-grade nodules, respectively. Eating wild cabbage 1–2 times daily and consuming radishes 1–6 times weekly increased the likelihood of developing higher-grade nodules by 5.72 and 4.98 times, respectively, compared to not eating them at all. Furthermore, individuals who consume disulfide-containing foods 1–6 times weekly and 1–2 times daily were 0.16 and 0.25 times more likely to have higher nodular grading than those who did not consume these foods at all, respectively.

**Table 6:** Logistic regression analysis of factors influencing the grade of thyroid nodules.

Variable	Level	β	SE	OR (95%CI)	P
Sex	Male			Ref	
	Female	2.579	0.71	13.19 (3.39–55.64)	<0.05
BMI	Underweight			Ref	
	Normal weight	-1.605	1.56	0.20 (0.01–3.77)	0.154
	Overweight	0.908	0.49	2.48 (0.95–6.65)	<0.05
	Obesity	1.355	0.55	3.88 (1.32–11.73)	<0.05
Smoking status	Nonsmoker			Ref	
	Smoker	1.578	0.82	4.84 (0.97–24.63)	<0.05
Salt intake	Moderate			Ref	
	Light	-0.637	0.43	0.53 (0.23–1.23)	0.071
	Heavy	-2.083	0.57	0.13 (0.04–0.37)	<0.05
Frequency of cabbage consumption	Never			Ref	
	1–6 times weekly	1.534	0.80	4.64 (0.98–23.19)	<0.05
	1–2 times daily	2.024	0.92	7.57 (1.26–48.24)	<0.05
Frequency of wild cabbage consumption	Never			Ref	
	1–6 times weekly	0.106	0.55	1.11 (0.38–3.30)	0.424
	1–2 times daily	1.743	0.95	5.72 (0.90–37.44)	<0.05
Frequency of eating radish	Never			Ref	
	1–6 times weekly	1.605	0.74	4.98 (1.19–21.82)	<0.05
	1–2 times daily	-0.243	1.09	0.78 (0.09–6.63)	0.412
Eating disulfide-containing foods	Never			Ref	
	1–6 times weekly	-1.823	0.84	0.16 (0.03–0.83)	<0.05
	1–2 times daily	-1.397	0.82	0.25 (0.05–1.24)	<0.05

**Figure 4:**
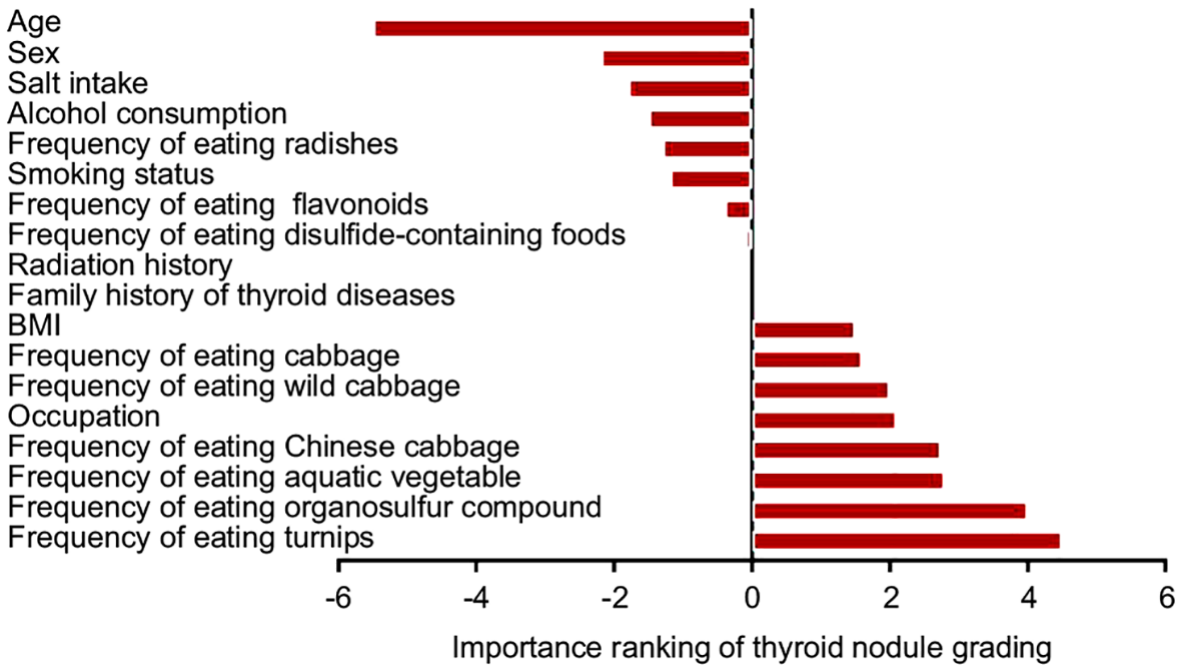
Importance ranking of factors influencing thyroid nodule grading.

Random forest verification results showed that the average Gini index of variables, such as age classification, sex, salt intake, and alcohol consumption in the past year, had a significant impact on nodular grading (**Figure 4**).

### 3.5 Analysis of factors influencing benign and malignant classification of TNs

#### 3.5.1 Demographic distribution of patients with benign and malignant TNs

A total of 116 participants were included in the analysis. Benign TNs were considered controls, and other factors were analyzed as independent variables. The demographic characteristics of the participants are shown in **Table 7**.

**Table 7:** Demographic characteristics of participants with benign and malignant thyroid nodules.

Variable	Level	Benign and malignant		p
		Benign (N=72)	Malignant (N=44)	
Urinary iodine (%)	Iodine deficiency	27 (37.5)	20 (45.5)	0.059
	Adequate	12 (16.7)	13 (29.5)	
	Iodine excess	33 (45.8)	11 (25.0)	
Sex (%)	Male	14 (19.4)	6 (13.6)	0.582
	Female	58 (80.6)	38 (86.4)	
Age (%)	18–30	8 (11.1)	4 (9.1)	0.953
	31–40	10 (13.9)	6 (13.6)	
	41–50	15 (20.8)	11 (25.0)	
	51–60	39 (54.2)	23 (52.3)	
BMI (%)	Underweight	22 (30.6)	8 (18.2)	0.235
	Normal weight	2 (2.8)	0 (0.0)	
	Overweight	33 (45.8)	22 (50.0)	
	Obesity	15 (20.8)	14 (31.8)	
Occupation (%)	Light labor	5 (6.9)	3 (6.8)	0.876
	Heavy labor	55 (76.4)	32 (72.7)	
	Other	12 (16.7)	9 (20.5)	
Family history of thyroid diseases (%)	No	69 (95.8)	44 (100.0)	0.442
	Yes	3 (4.2)	0 (0.0)	
Radiation history (%)	No	69 (95.8)	43 (97.7)	0.986
	yes	3(4.2)	1 (2.3)	
Alcohol consumption (%)	Non-drinker	58 (80.6)	38 (86.4)	0.582
	Drinker	14 (19.4)	6 (13.6)	
Smoking status (%)	Nonsmoker	67 (93.1)	39 (88.6)	0.63
	Smoker	5 (6.9)	5 (11.4)	
Salt intake (%)	Moderate	25 (34.7)	19 (43.2)	0.015
	Light	26 (36.1)	22 (50.0)	
	Heavy	21 (29.2)	3 (6.8)	

#### 3.5.2 Factors influencing the classification of TNs

After controlling for urinary iodine, the probability of developing malignant nodules in women was 8.18 times that in men. Eating radishes 1–6 times weekly was 26.19 times more likely to result in a malignant nodule than not eating them at all. Those who consumed turnips 1–6 times weekly were 27.84 times more likely to develop malignant nodules than those who did not eat them at all. A diet with heavy salt intake was 0.10 times less likely to result in a malignant nodule than a moderate diet (**Table 8**).

**Table 8:** Logistic regression analysis of factors associated with benign and malignant thyroid nodules.

Variable	Level	Crude OR (95%CI)	Adjusted OR (95%CI)
Sex	Male	Ref	Ref
	Female	5.72 (0.87–37.74)	8.18 (1.13–59.38)*
Smoking status (%)	Nonsmoker	Ref	Ref
	Smoker	6.73 (0.70–64.81)	8.56 (0.79–92.32)
Salt intake (%)	Moderate	Ref	Ref
	Light	0.93 (0.36–2.42)	0.91 (0.34–2.45)
	Heavy	0.12 (0.02–0.59) *	0.10 (0.02–0.54) *
Frequency of eating radish	Never	Ref	Ref
	1–6 times weekly	20.22 (1.67–245.33) *	26.19 (2.12–323.67) *
	1–2 times daily	4.72 (0.18–127.17)	5.23 (0.18–148.18)
Frequency of eating turnip	Never	Ref	Ref
	1–6 times weekly	19.31(1.49–249.82) *	27.84 (1.78–435.20) *
	1–2 times daily	169111575.31 (0.00–Inf)	133099357.96 (0.00–Inf)

Model adjusted for urinary iodine levels; *P<0.05.

**Figure 5:**
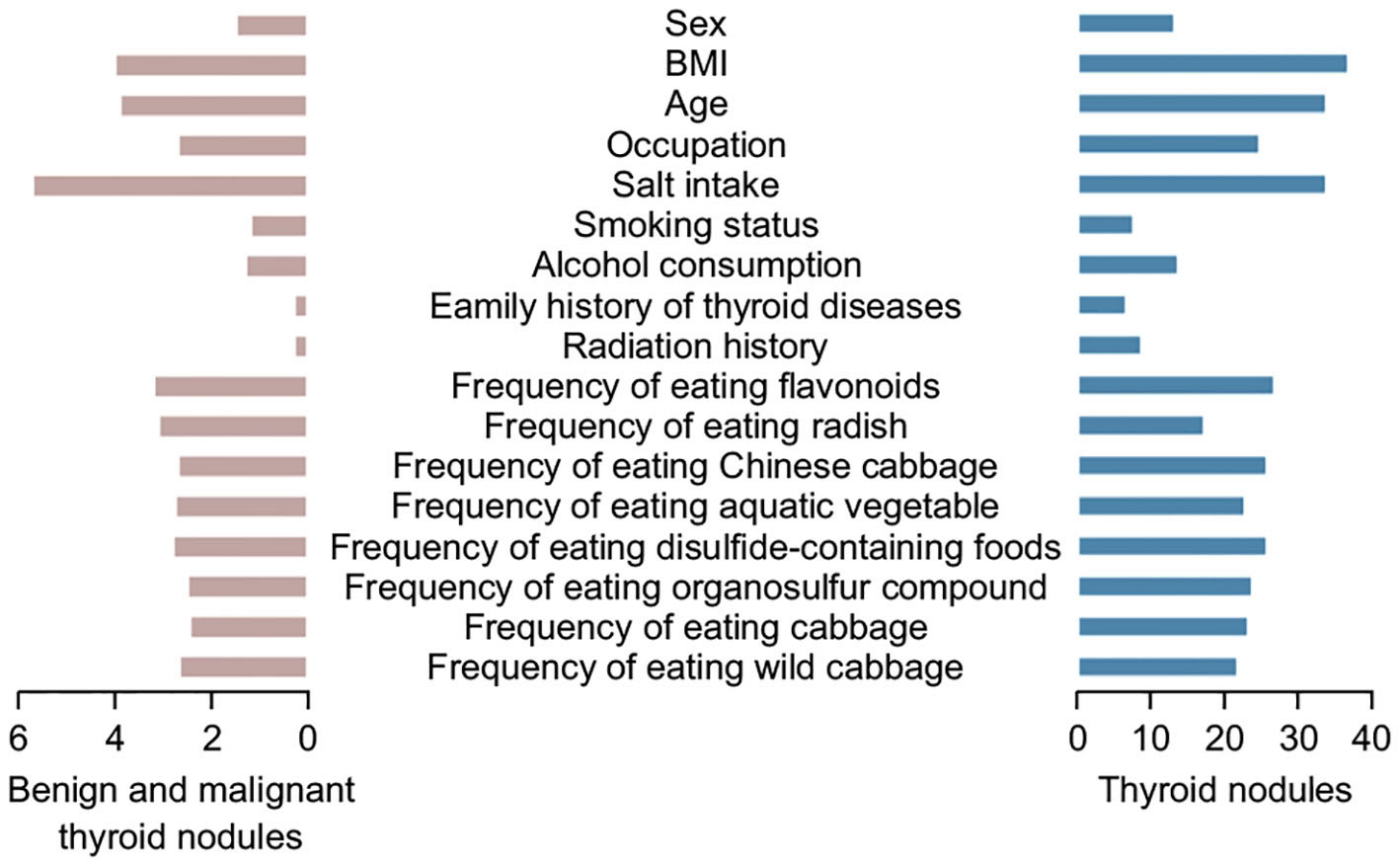
Ranking of important factors influencing the development of benign and malignant (left) thyroid nodules and all thyroid nodules (right).

For the risk of developing benign or malignant TNs, the random forest model was used to rank the importance of the meaningful independent variables. According to the average Gini decline index (**Figure 5**), BMI classification, age, and salt intake had a significant impact on both aspects.

**Figure 6:**
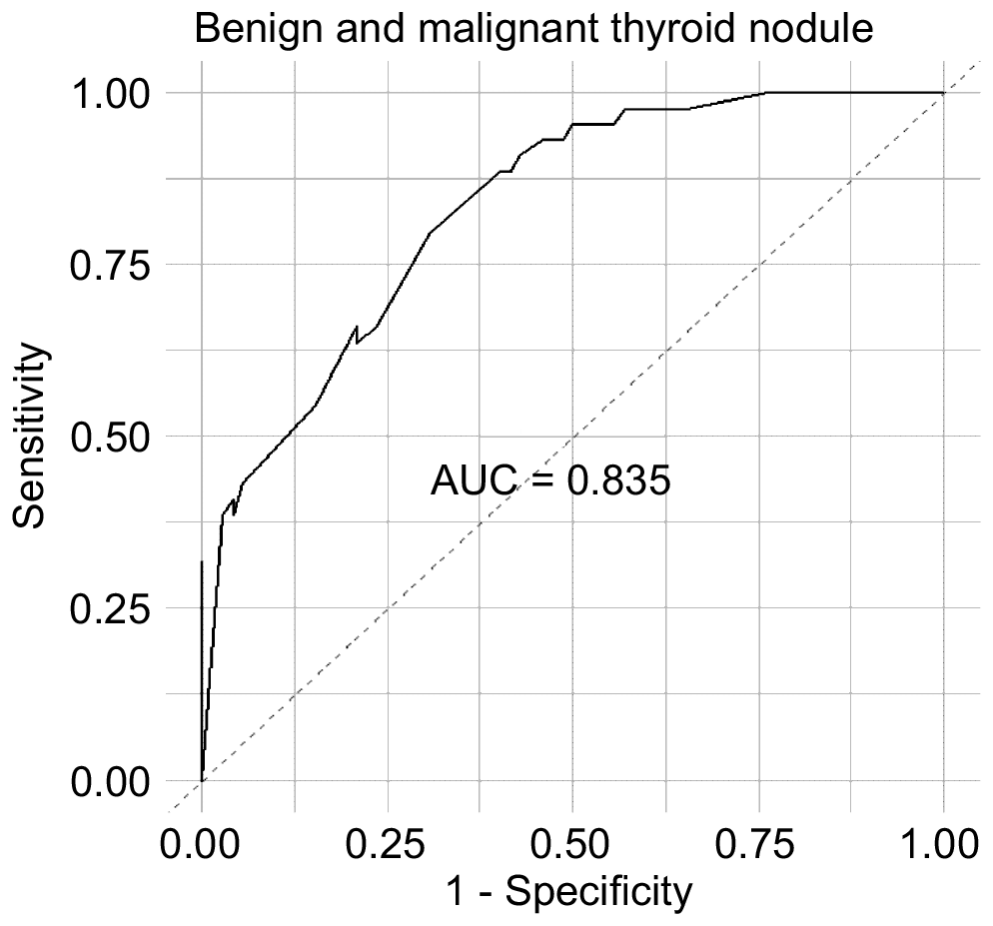
ROC curves of variables affecting benign and malignant nodules.

The ROC curve was drawn for the results of the stepwise regression model, revealing an AUC of 0.835, indicating that the model had a good predictive ability for differentiating between benign and malignant nodules. The stepwise regression analysis identified urinary iodine, sex, salt intake, and the frequency of radish and turnip consumption as significant factors influencing TN, indicating their crucial role in predicting the presence or absence of nodules (**Figure 6**).

## 4 Discussion

This cross-sectional study investigated the prevalence of TNs in a population of 1,697 adults residing in Anhui Province and analyzed the risk factors that may predispose individuals to develop TNs. The benign and malignant trends of TNs and their risk factors were evaluated by grading the TNs, offering insight into the early identification of malignant thyroid tumors. Our findings revealed that TNs are a common thyroid disease in adults, with a significantly higher detection rate among women than among men. Moreover, the prevalence of nodules in our study tended to increase with age, consistent with the results from cross-sectional studies conducted in 30 provinces and cities in China (20).

The prevalence of TNs in adults can be as high as 50%, which increases with age and is more prevalent in women and smokers (21). Estrogen regulates thyrotropin production, with estrogen receptor expression in thyroid cells influencing cell growth and nodular formation of the thyroid, resulting in a higher prominence of TNs in women than in men (22). The findings in our study further confirmed the higher prevalence of TNs in women, with an overall prevalence of 24.5% in women and 13.2% in men, and that women were 1.67 times more likely to develop nodules than men. In addition, the possibility of TNs deterioration in females was higher than that in males. When other independent variables remained unchanged, the proportion of graded nodule deterioration in females increases by 13.19 times compared with males, with the risk of nodules increasing in older age groups. This finding is consistent with the results of other studies (9, 23, 24). Studies have suggested that the high prevalence of TNs in older individuals is related to a decline in thyroid function due to aging. With advancing age, thyroid tissue undergoes fibrosis, cell infiltration, and follicle-like changes, eventually forming nodules (25).

In this study, the prevalence of TNs was higher in the overweight and obese groups than that in the normal body weight group. The relationship between BMI and TNs may be related to leptin secreted by the adipose tissue. Elevated serum leptin concentrations in obese individuals can promote an increase in thyrotropin levels, thus contributing to the onset and progression of TNs (26).

Our findings revealed that smokers were 4.84 times more likely to have higher nodule grading than non-smokers. This association may be attributed to nicotine, thiocyanate, and other components of tobacco. Thiocyanate competitively inhibits the activity of peripheral deiodinase by blocking the organic process of iodine absorption and inhibition, accelerating iodine efflux and iodinated tyrosine coupling, and increasing serum thyroid-stimulating hormone (TSH) expression. This cascade ultimately promotes the formation of TNs (27).

Our results showed that alcohol consumption is a protective factor against the development of TNs. Although the specific mechanism through which this occurs remains unclear, it may involve the inhibition of thyroid hormone metabolism by alcohol, increased thyroid cell sensitivity to TSH, inhibition of thyroid cell proliferation, and direct toxic effects on the thyroid gland (28).

Iodine intake is suspected to be a major dietary factor affecting the development. Participants with high or excessive iodine intake did not demonstrate an increased risk of TNs, whereas insufficient iodine intake was significantly associated with a higher risk of nodules (29). To account for the influence of iodine, we adjusted for urinary iodine levels in each part of the study. Interestingly, several studies have investigated the presence of thyroxine in food and found that it is important in certain geographic areas with endemic diseases (e.g., TN and goiter) (30).

Currently, high iodine levels are known to directly damage thyroid follicular cells and increase the occurrence of TNs by binding with thyroglobulin to become iodized thyroglobulin (31). This process enhances the immunogenicity of thyroglobulin, leading to the presentation of antigen peptides and an increase in the number of pathogenic T lymphocytes. It can also increase the aggression of immune cells and phagocyte function by inducing the abnormal expression of major histocompatibility complex class II. Furthermore, goiter is clinically recognized to be caused by iodine deficiency. The incidence of TNs in high iodine states is believed to be lower than that for other thyroid diseases, although no clear conclusions exist on this incidence compared with what is observed in an adequate iodine state.

An individual’s iodine intake is influenced by a variety of factors, including the environmental iodine concentration, dietary habits, and absorption capacity. Environmental iodine concentration levels vary greatly in different regions of China, and thyroid disease prevention can be achieved when medina urinary iodine concentrations are maintained between 100 and 299 µg/L. According to a special report, the median urine iodine concentration of adults in 24 provinces and cities in China is 180.3 μg/L (31). Iodine nutritional status was defined using the values obtained from the general population. However, it should be noted that this study had a cross-sectional design and could not directly establish a causality between iodine intake and TNs.

Based on the factors we analyzed, we found that people who regularly consumed cabbage, radishes, and wild cabbage had a higher likelihood of developing malignant nodules. These foods may be a risk factor for TNs becoming malignant. However, in recent years, more and more studies have shown that isothiocyanates and flavonoids in these vegetables can effectively prevent the occurrence and development of various tumors (31). These compounds have been shown to reduce the risk of various tumors, including lung, stomach, colon, and liver cancers, by effectively preventing cancer cell deterioration, inhibiting metastasis, and controlling the spread of cancer cells. Importantly, isothiocyanates and flavonoids have demonstrated efficacy in preventing the growth of thyroid cancer cells and inhibiting the proliferation of human thyroid cancer cell lines and primary cells, suggesting a potential anti-thyroid cancer effect (32).

As the risk of developing malignant tumors from TNs is not yet clear, our study sought to investigate the detection rate and risk factors of TNs across different urinary iodine regions in Anhui, China, through a population survey. We stratified the benign and malignant trends of TNs by risk stratification and analyzed the risk factors for these nodules using questionnaire responses. This approach can assist in guiding the evaluation of benign and malignant TNs and inform treatment decisions, which are crucial for the early diagnosis and prevention of malignant thyroid tumors (33). Iodine intake affects the score of thyroid disease, and small changes in iodine intake in a population can affect the risk of thyroid disease. Consistent with previous findings (34), this study found that the prevalence of goiter decreased with the increase of iodine intake. However, this study found that the prevalence of TNs was higher in regions with different iodine intake, which was consistent with previous analysis (35).

Our study has some limitations, such as sample size and sampling technique. Hence, more extensive epidemiological investigations and cohort studies are required to analyze the risk factors involved in TNs pathogenesis. In addition, controlling habits and dietary iodine intake poses challenges. The fast-paced lifestyle prevalent in today’s society contributes to increased stress levels, which are also significant contributors to thyroid disease. This study found that increased iodine intake was associated with a lower prevalence of TNs. Nevertheless, further cohort studies are needed to explore the potential causes of the high prevalence of TNs.

## 5 Conclusions

The ultrasonographic detection rate of TNs in the five regions of the Anhui Province was 20.9%. Factors such as female sex, older age, obesity and smoking are risk factors for both the development and deterioration of TNs. Consumption of vegetables rich in sulfides and flavonoids was found to be a protective factor against TNs disease, although this increased the likelihood of deterioration in those already diagnosed with TNs. Therefore, patients with TNs should exercise caution when consuming these vegetables and adhere to a scientifically balanced diet to prevent development of TNs. Given the high incidence of TNs in our setting, raising public health awareness is critical among residents, and regular thyroid ultrasound screening is necessary to facilitate early detection and treatment.

## Data Availability

The original contributions presented in the study are included in the article/**Supplementary Material**, Further inquiries can be directed to the corresponding author/s.
