# Supplementary material for: Analysis of factors influencing prevalence and malignancy of thyroid nodules in various iodine uptake areas
Source: Front Endocrinol (Lausanne). 2024 Nov 7;15:1451911. doi: 10.3389/fendo.2024.1451911 (PMC11578705; doi:10.3389/fendo.2024.1451911)
Supplement: Supplementary file 1 [file DataSheet1.docx]

Supplementary Material

# Supplementary Figures


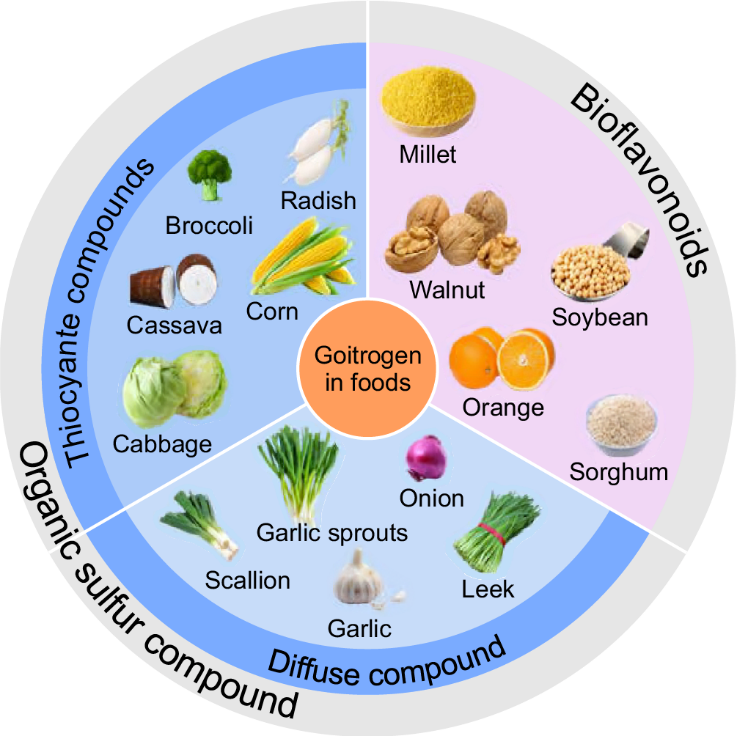


**Supplementary Fig. 1** Classification of goitrogenic substances in food


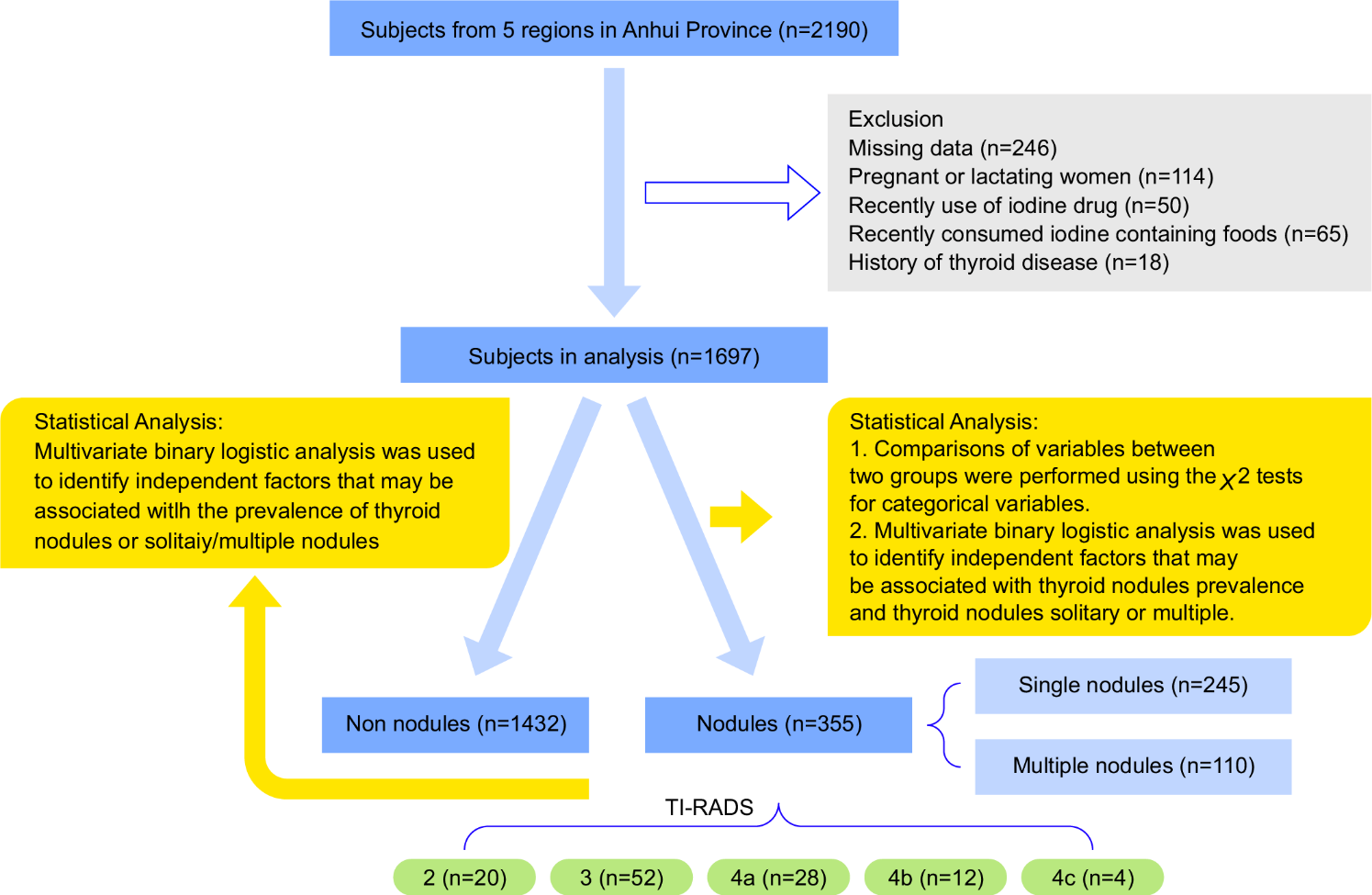


**Supplementary Fig. 2** Flowchart showing the inclusion and exclusion of participants

**
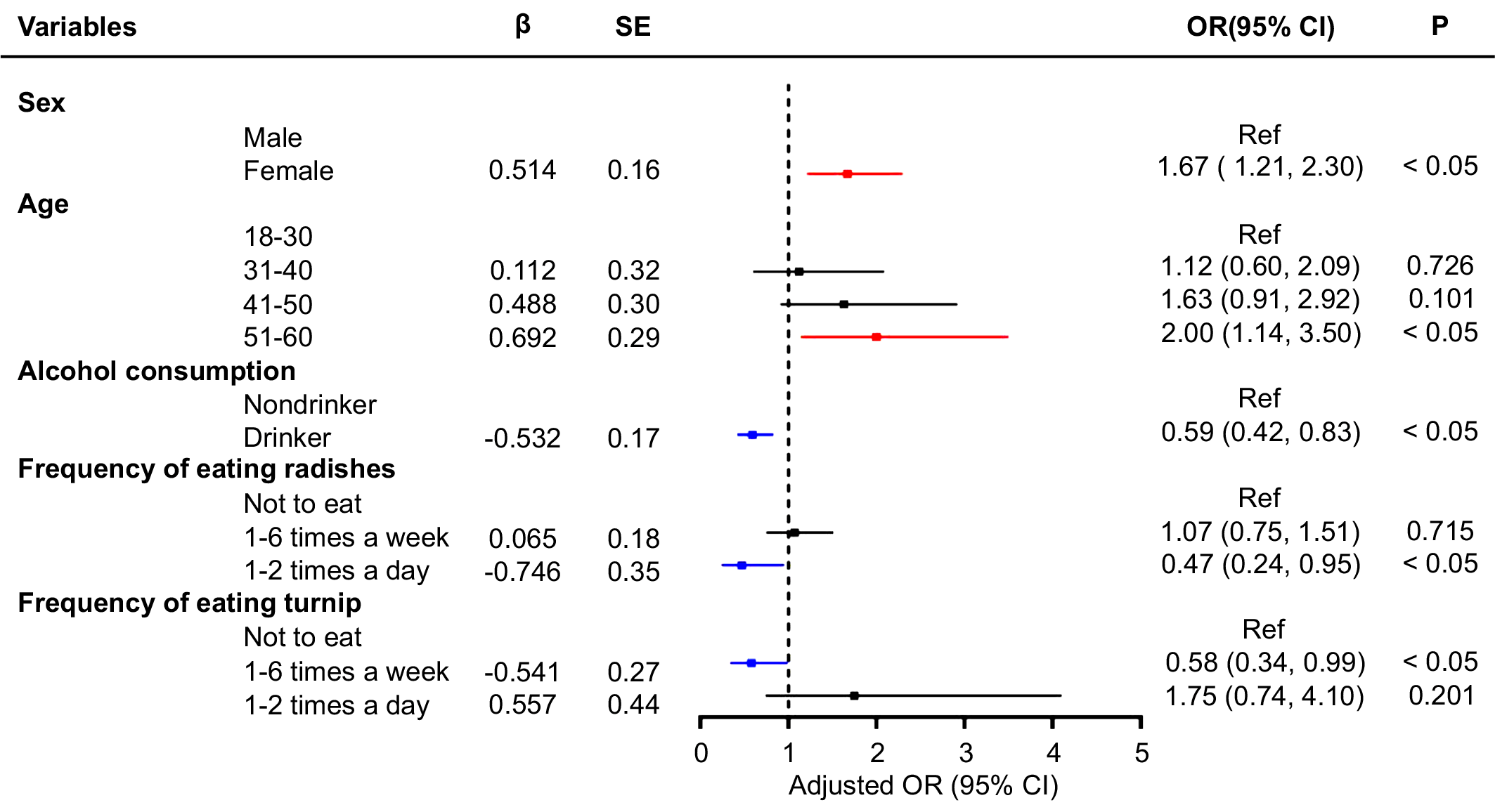
**

**Supplementary Fig. 3** Multivariate logistic regression analysis of factors associated with thyroid nodules

SE, standard error; OR, odds ratio; CI, confidence interval

**
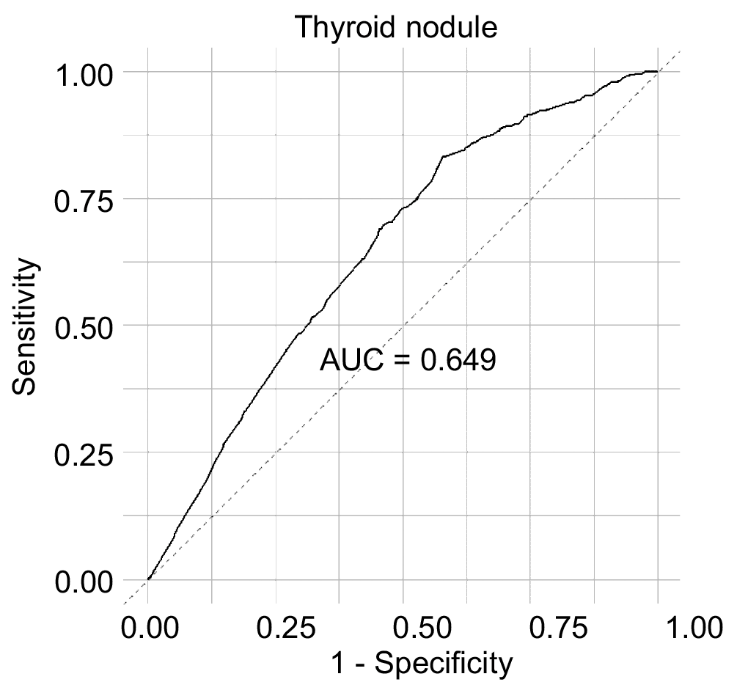
**

**Supplementary Fig. 4** Receiver operating characteristic (ROC) curve of variables that predict the occurrence of thyroid nodules. AUC, area under the curve
